# Supplementary material for: Data-driven predictions of complex organic mixture permeation in polymer membranes
Source: Nat Commun. 2023 Aug 15;14:4931. doi: 10.1038/s41467-023-40257-2 (PMC10427679; doi:10.1038/s41467-023-40257-2)
Supplement: Supplementary file 1 — Supplementary Information [file 41467_2023_40257_MOESM1_ESM.pdf]

Supplementary information for

Data-driven predictions of complex organic mixture permeation in polymer membranes

Young Joo Lee,[†] Lihua Chen,[†] Janhavi Nistane, Hye Youn Jang, Dylan J. Weber, Joseph K. Scott, Neel D. Rangnekar, Bennett D. Marshall, Wenjun Li, J.R. Johnson, Nicholas C. Bruno, M.G. Finn, Rampi Ramprasad,* Ryan P. Lively*

Correspondence to: rampi.ramprasad@mse.gatech.edu, ryan.lively@chbe.gatech.edu

This file includes:

Supplementary Figures 1 to 7

Supplementary Tables 1 to 15

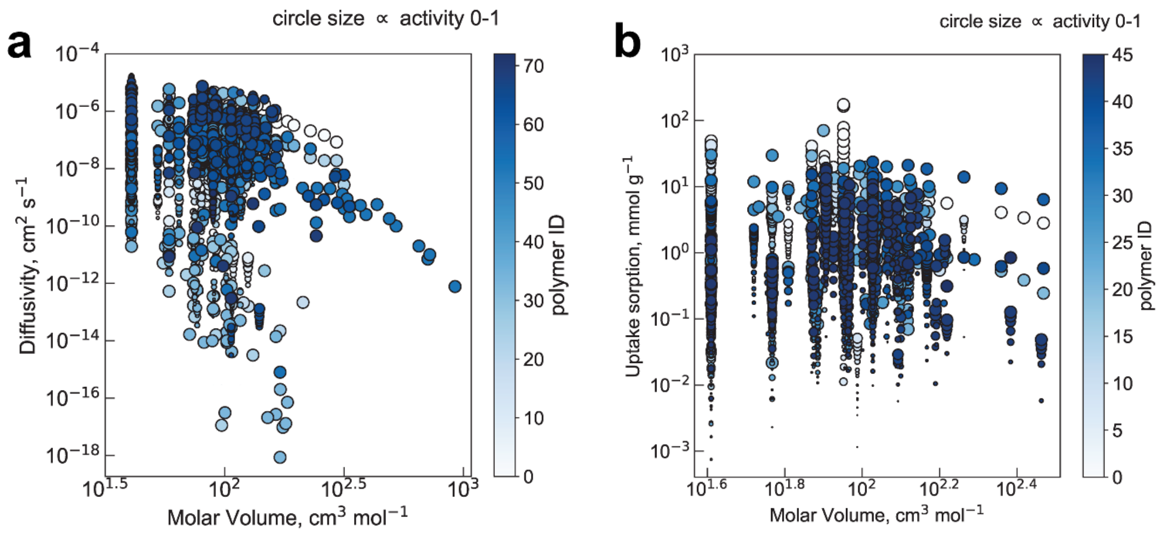

Supplementary Figure 1.

Overview of the database that has been used to make machine learning (ML) models: (a) Fickian diffusion coefficients ($cm^2\ s^{-1}$) and (b) sorption uptakes ($mmol\ g^{-1}$). Thermodynamic activities (vapor pressure over saturation vapor pressure at a given temperature and unit activity in cases of liquid sorption) are described by circle sizes in the plots - the certain activity values of each data points in the plots are provided in the Source Data file. Every polymer ID represents a unique polymer in the dataset, and the IDs are assigned arbitrarily. Source data are provided in the Source Data file.

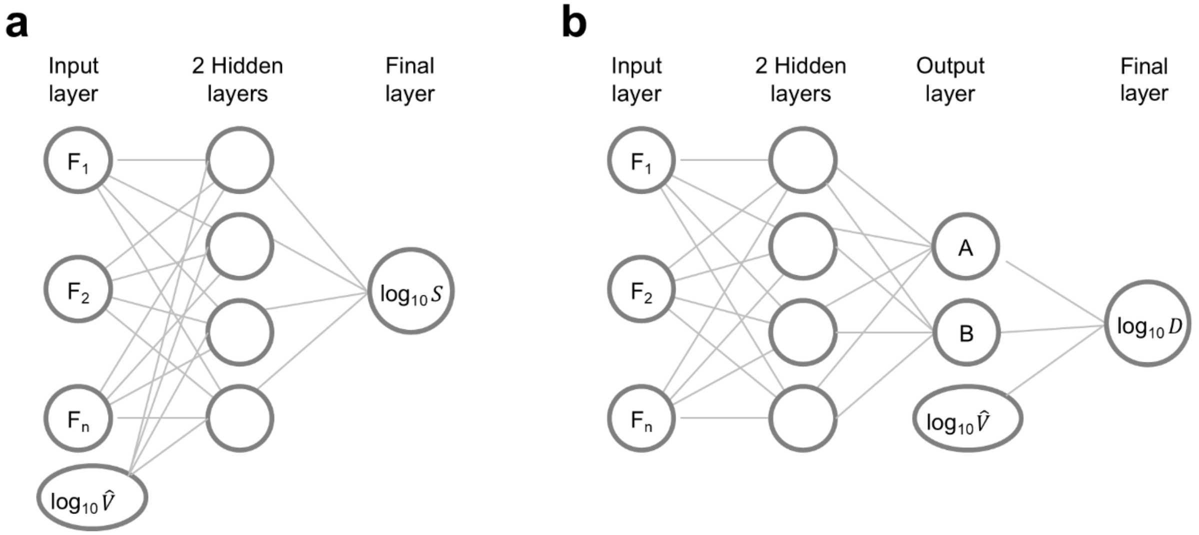

Supplementary Figure 2.

Machine learning (ML) methods. The architecture of (a) traditional deep neural network (NN) model for sorption and (b) physics-informed NN model for diffusion. Both models include an input layer, 2 hidden layers, and a final layer of $\log_{10} S$ or $\log_{10} D$ for estimating loss function, Here, S and $\log_{10} D$ indicates the target sorption uptakes (mmol g^{-1}) and Fickian diffusion coefficients (cm^2 s^{-1}), respectively. The F_n features within the input layer represent chemical features of polymers, solvents, and experimental activities. $\hat{V}$ denotes the molar volume of solvents, which is one of the features of the input layer. In case of the ML diffusion model (b), an additional output layer was introduced to train A and B parameters in $\log_{10} D = A \cdot \log_{10} \hat{V} + B$ using the F_n features. The output layer was followed by estimating $\log_{10} D$ using $\log_{10} \hat{V}$ and the physical equation ($\log_{10} D = A \cdot \log_{10} \hat{V} + B$) to enforce the NN models to learn the physical relationship. The physics-informed ML diffusion model uses the trained A and B parameters to calculate the target property, D.

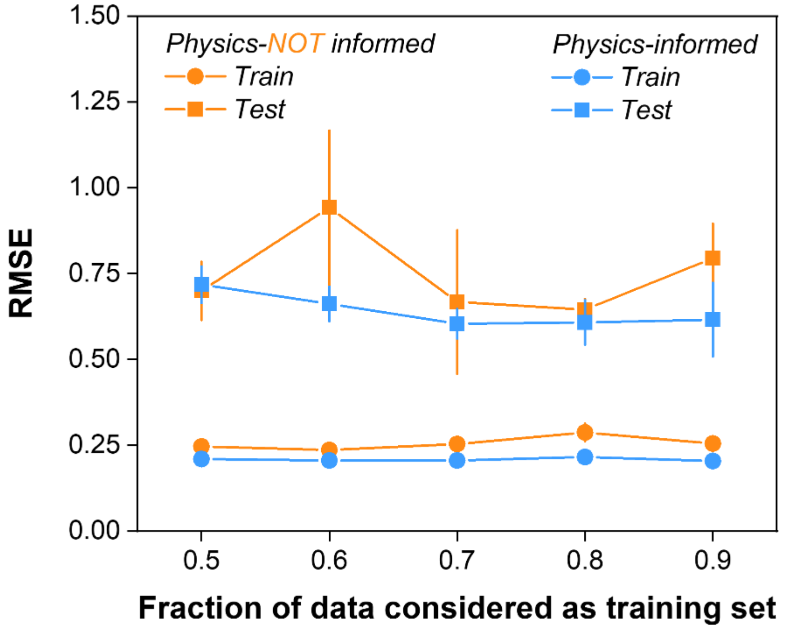

Supplementary Figure 3.

Learning curves of two machine learning (ML)-diffusion models before and after infusing the size-based physics. The architecture of the neural network (NN) model without the physics infusion is the same as the sorption model shown in the Supplementary Fig. 2a. In this plot, the learning curves describe the RMSE (root mean square error, Eq. (16)) variation of training/test sets as a function of training set size (e.g., fraction of data considered in model training). Error bars in the plot were obtained from 10 predictions from 10-fold cross-validation steps. Source data are provided in the Source Data file.

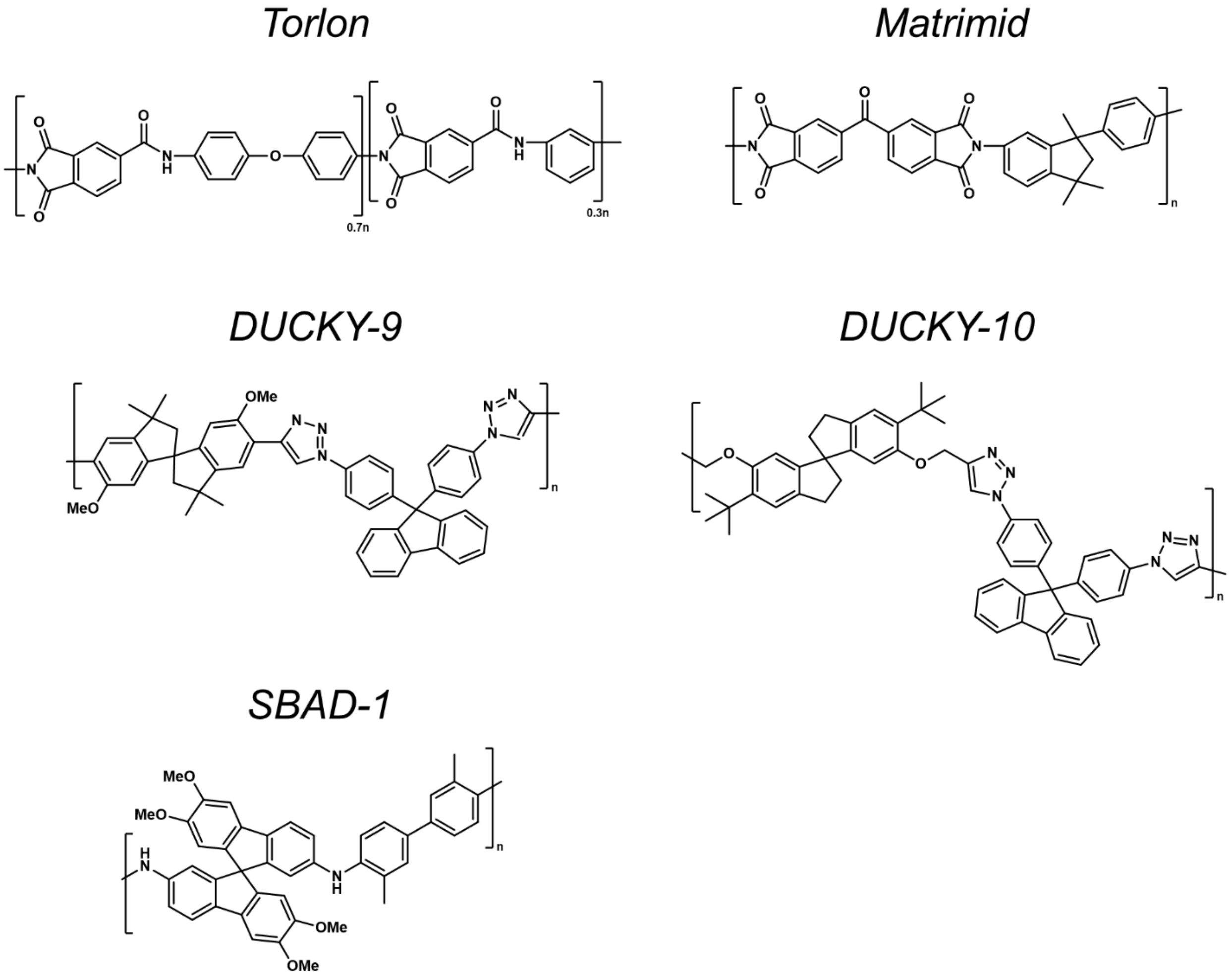

Supplementary Figure 4.

Chemical structures of polymers that were tested in the separations of 12- and 9-component hydrocarbon mixtures (Fig. 3) and two crude oil mixtures (Fig. 4).

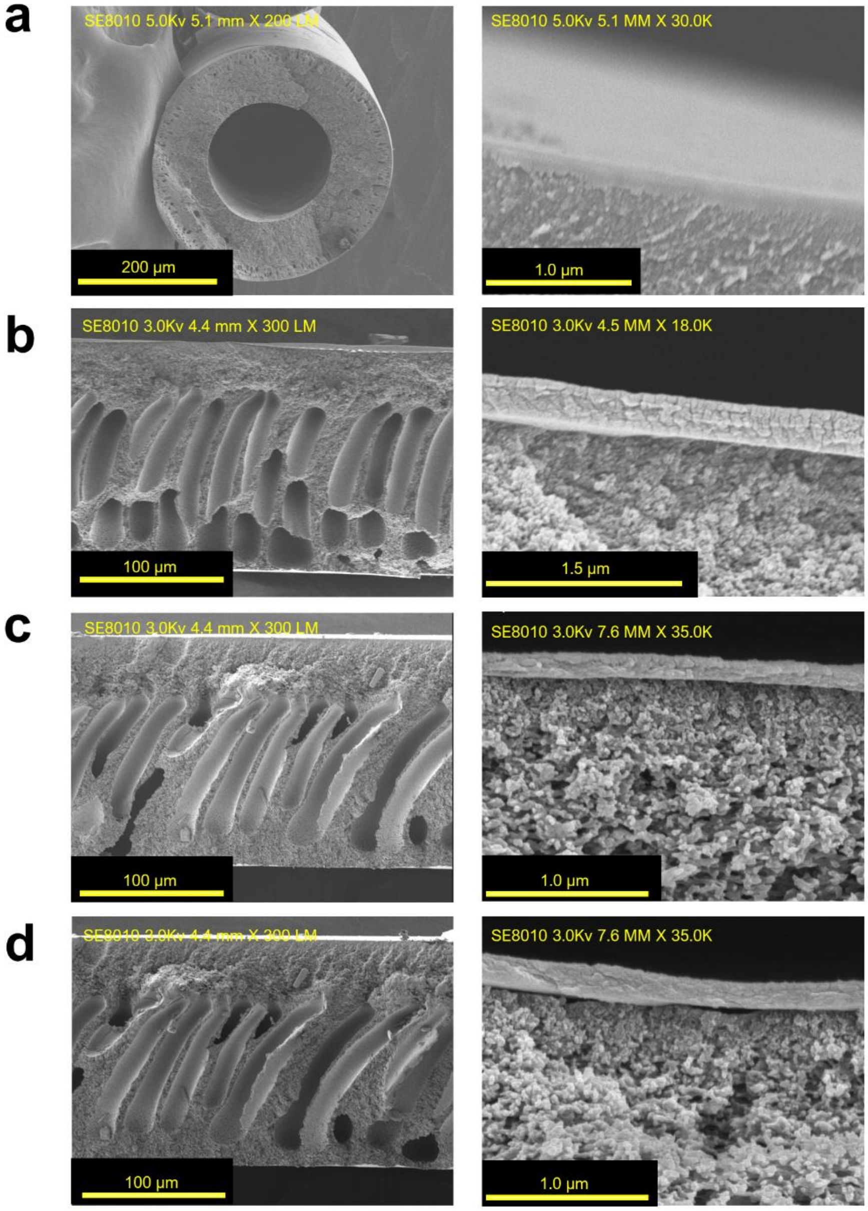

Supplementary Figure 5.

Cross-sectional Scanning Electron Microscopy (SEM) images. From the top to bottom: (a) Torlon, (b) Matrimid, (c) DUCKY-9, and (d) DUCKY-10 membrane. The skin layer thickness was determined using calibrated distance estimates in the SEM software. In the predictions, 100 nm, 350 nm, 150 nm, and 200 nm were assumed for Torlon, Matrimid, DUCKY-9, and DUCKY-10, respectively.

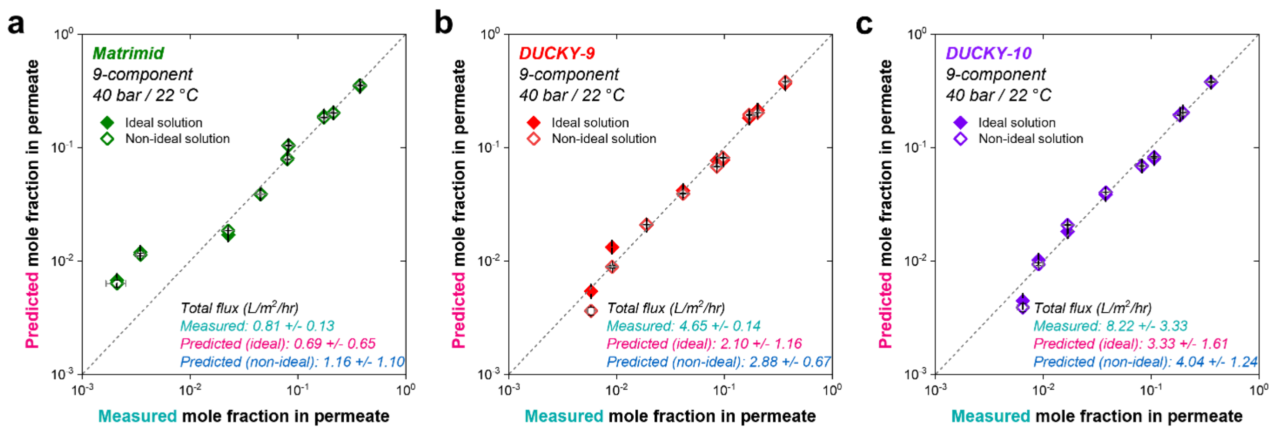

Supplementary Figure 6.

Validation of ideal solution assumption in predicting hydrocarbon mixture separations (**a**-Matrimid, **b**-DUCKY-9, **c**-DUCKY-10). Comparison between hydrocarbon mixture separation predictions with ideal solution assumption and with non-ideal solution assumption. All experiments and predictions were performed at 22 °C. The pressures in the plots indicate the transmembrane pressure (i.e., the applied pressure at the upstream side of the membrane minus the atmospheric pressure present on the membrane downstream). The error bars represent the standard deviation of the permeate concentration predictions for each molecule, and the deviations are from uncertainty in the machine learning (ML) sorption model parameter predictions. The deviations in the total flux predictions are from the uncertainty in the ML diffusion model predictions (Supplementary Tables 4-6). PC-SAFT in ASPEN plus was used in the activity coefficient calculation (Supplementary Tables 8-10). The use of non-ideal activity coefficients in the prediction resulted in slightly higher flux predictions that exhibited better agreement with the experimental measurements. Furthermore, the predicted permeate concentrations showed improve accuracy compared to the measured permeate concentrations, as demonstrated by lower RMSPE and AOME values (Supplementary Tables S8-10). Source data are provided in the Source Data file.

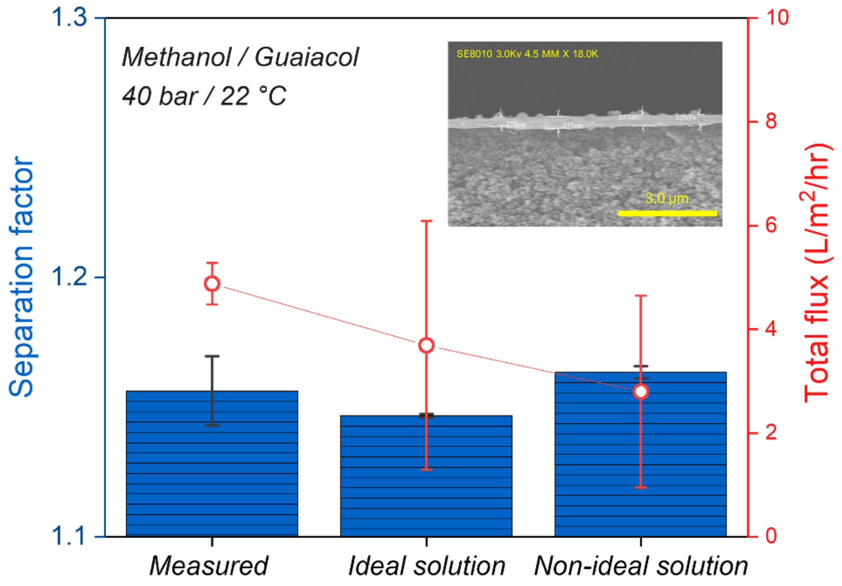

Supplementary Figure 7.

Separation of a binary mixture of oxygenated molecules as a bio-fuel type mixture via DUCKY-9 membrane. The separation factors of guaiacol and total fluxes are indicated by bars and red circles, respectively. The feed mixture in this crossflow permeation experiment was 80 mol% of methanol and 20 mol% of guaiacol. The test was conducted at 40 bar transmembrane pressure and 22 °C. The diffusivities and solubilities were predicted by the machine learning (ML) models, and the experimental operating conditions were applied in the transport modeling as described in Methods section in the main article. A 300 nm membrane thickness was determined from a scanning electron microscopy (SEM) image of one of the tested DUCKY-9 membranes (inset). The model estimates and solvent properties are summarized in Supplementary Tables 11-12, and the separation factors here were calculated by Eq. (17). The error bars in the measured separation factor and total flux are from the results of two DUCKY-9 membrane tests. The error bars in separation factor predictions are from the uncertainty in the ML sorption parameter predictions, and the deviations in the total flux predictions are from the uncertainty in the ML diffusion parameter predictions. SR-POLAR model in ASPEN plus was used in the activity coefficient calculation for the non-ideal mixture-based predictions (Supplementary Table 13). Source data are provided in the Source Data file.

Supplementary Table 1.

Details of experimental diffusivity and sorption uptake datasets, number of features, and optimized parameters of final prediction models in the Supplementary Fig. 2. Features contain chemical features of polymers and solvents, thermodynamic activities and molar volumes. The final prediction models were trained using the entire datasets and were validated by 10-fold cross-validation (CV).

		Diffusivity	Sorption uptakes
Datasets (experimental values)		2045 (73 polymers+151 solvents)	2275 (46 polymers+91 solvents)
Features	Chemical features	440	368
	Activities	1	1
	Molar volume	1	1
Optimized parameters within NN models	Number of neurons with 2 hidden layers	256	128
	activation function	sigmoid	sigmoid
	learning rate	0.01	0.001
	drop out	0.02	0.01

Supplementary Table 2.

Feed concentrations and solvent properties. From top to bottom: Torlon, Matrimid, DUCKY-9, DUCKY-10. δ_D, δ_P, and δ_H are Hansen solubility parameters for dispersion, polarity, and hydrogen-bonding each.

Torlon	Concentration	Hansen solubility parameter ($MPa^{0.5}$)			Vapor pressure (torr)	Molar volume (cm^3 mol^{-1})
Component	Mole fraction	δ_D	δ_P	δ_H		
toluene	0.239	18.0	1.4	2.0	29.99	106.52
o-xylene	0.05	18.0	2.8	3.0	6.70	121.76
propylbenzene	0.22	17.4	2.2	2.8	2.94	138.81
mesitylene	0.08	17.8	2.6	3.0	1.76	138.29
n-butylcyclohexane	0.08	16.4	0	0	0.95	176.71
Tetralin	0.15	18.6	2.2	2.9	0.51	140.13
1-methylnaphthalene	0.04	20.6	0.8	4.7	2.06	139.82
biphenyl	0.04	19.3	2.3	3.3	0.04	149.89
1,3,5-triisopropylbenzene	0.08	18.0	0	0.6	0.04	240.07
dodecylbenzene	0.01	16.8	1.1	1.8	$2.4\cdot10^{-4}$	283.30
1,3,5-triphenylbenzene	0.01	19.9	1.4	2.5	$4.1\cdot10^{-8}$	275.04
1,3,5-Tris[(3-methylphenyl)phenylamino]benzene	0.001	19.8	1.5	4.5	$2.5\cdot10^{-14}$	507.65

Matrimid	Concentration	Hansen solubility parameter ($MPa^{0.5}$)			Vapor pressure (torr)	Molar volume (cm^3 mol^{-1})
Component	Mole fraction	δ_D				
iso-octane	0.117	14.1	0	0	49.08	163.42
n-octane	0.188	15.5	0	0	14.80	165.55
methylcyclohexane	0.197	16.0	0	1.0	46.60	128.12
toluene	0.327	18.0	1.4	2.0	29.99	106.52
decalin	0.089	18.0	0	0	0.97	156.96
tert-butylbenzene	0.039	17.4	0.1	1.1	2.11	155.53
iso-cetane	0.011	16.3	0	0	0.05	293.27
1,3,5-triisopropylbenzene	0.014	18.0	0	0.6	0.04	240.07
1-methylnaphthalene	0.016	20.3	0.8	4.7	2.06	139.82

DUCKY-9	Concentration	Hansen solubility parameter ($MPa^{0.5}$)			Vapor pressure (torr)	Molar volume (cm^3 mol^{-1})
Component	Mole fraction	δ_D	δ_P	δ_H		
iso-octane	0.113	14.1	0	0	49.08	163.42
n-octane	0.170	15.5	0	0	14.80	165.55
methylcyclohexane	0.202	16.0	0	1.0	46.60	128.12
toluene	0.325	18.0	1.4	2.0	29.99	106.52
decalin	0.098	18.0	0	0	0.97	156.96
tert-butylbenzene	0.041	17.4	0.1	1.1	2.11	155.53
iso-cetane	0.014	16.3	0	0	0.05	293.27
1,3,5-triisopropylbenzene	0.018	18.0	0	0.6	0.04	240.07
1-methylnaphthalene	0.018	20.3	0.8	4.7	2.06	139.82

DUCKY-10	Concentration	Hansen solubility parameter ($MPa^{0.5}$)			Vapor pressure (torr)	Molar volume (cm^3 mol^{-1})
Component	Mole fraction	δ_D	δ_P	δ_H		
iso-octane	0.117	14.1	0	0	49.08	163.42
n-octane	0.187	15.5	0	0	14.80	165.55
methylcyclohexane	0.194	16.0	0	1.0	46.60	128.12
toluene	0.336	18.0	1.4	2.0	29.99	106.52
decalin	0.087	18.0	0	0	0.97	156.96
tert-butylbenzene	0.037	17.4	0.1	1.1	2.11	155.53
iso-cetane	0.011	16.3	0	0	0.05	293.27
1,3,5-triisopropylbenzene	0.014	18.0	0	0.6	0.04	240.07
1-methylnaphthalene	0.016	20.3	0.8	4.7	2.06	139.82

Supplementary Table 3.

Transport parameters of 12 components in the tested mixture in Torlon polymer predicted by the machine learning (ML) models: sorption uptakes at unit activity (mmol g^{-1}) and Maxwell-Stefan diffusion coefficients ($Đ_i^{v,m}$, cm^2 s^{-1}) by thermodynamically correcting Fickian diffusion coefficients ($D_i^{v,m}$, cm^2 s^{-1}) predicted by the ML models at unit activity of each component in Torlon polymer (Eq. (12)). The lowest, average, and highest value were from the uncertainty of the ML predictions. The Flory-Huggins interaction parameters of single component at unit activity were calculated by Eq. (6) and Eq. (7).

Component	Lowest sorption (mmol g^{-1})	Sorption in average (mmol g^{-1})	Highest sorption (mmol g^{-1})	Lowest diffusion ($Đ_i^{v,m}$, cm^2 s^{-1})	Diffusion in average ($Đ_i^{v,m}$, cm^2 s^{-1})	Highest diffusion ($Đ_i^{v,m}$, cm^2 s^{-1})
toluene	0.493	0.656	0.872	$1.40 \cdot 10^{-9}$	$1.98 \cdot 10^{-9}$	$2.81 \cdot 10^{-9}$
o-xylene	0.714	0.865	1.048	$1.08 \cdot 10^{-9}$	$1.50 \cdot 10^{-9}$	$2.08 \cdot 10^{-9}$
propylbenzene	0.523	0.685	0.897	$6.7 \cdot 10^{-10}$	$9.80 \cdot 10^{-10}$	$1.39 \cdot 10^{-9}$
mesitylene	0.455	0.632	0.875	$6.66 \cdot 10^{-10}$	$9.32 \cdot 10^{-10}$	$1.30 \cdot 10^{-9}$
n-butylcyclohexane	0.516	0.630	0.768	$8.60 \cdot 10^{-12}$	$2.81 \cdot 10^{-11}$	$9.17 \cdot 10^{-11}$
Tetralin	0.086	1.457	2.444	$4.43 \cdot 10^{-11}$	$2.54 \cdot 10^{-10}$	$1.45 \cdot 10^{-9}$
1-methylnaphthalene	0.422	0.602	0.859	$4.87 \cdot 10^{-12}$	$5.81 \cdot 10^{-11}$	$6.94 \cdot 10^{-10}$
biphenyl	0.272	0.443	0.721	$9.90 \cdot 10^{-13}$	$1.49 \cdot 10^{-11}$	$2.24 \cdot 10^{-10}$
1,3,5-triisopropylbenzene	0.440	0.701	1.116	$9.17 \cdot 10^{-11}$	$1.29 \cdot 10^{-10}$	$1.83 \cdot 10^{-10}$
dodecylbenzene	0.446	0.610	0.834	$2.15 \cdot 10^{-11}$	$7.07 \cdot 10^{-11}$	$2.32 \cdot 10^{-10}$
1,3,5-triphenylbenzene	0.107	0.232	0.503	$5.64 \cdot 10^{-18}$	$1.46 \cdot 10^{-16}$	$3.78 \cdot 10^{-15}$
1,3,5-Tris[(3-methylphenyl)phenylamino]benzene	0.015	0.045	0.131	$5.13 \cdot 10^{-18}$	$7.62 \cdot 10^{-16}$	$1.13 \cdot 10^{-13}$

Supplementary Table 4.

Transport parameters of 9 components in the tested mixture in Matrimid polymer predicted by the machine learning (ML) models: sorption uptakes at unit activity (mmol g^{-1}) and Maxwell-Stefan diffusion coefficients ($Đ_i^{v,m}$, cm^2 s^{-1}) by thermodynamically correcting Fickian diffusion coefficients ($D_i^{v,m}$, cm^2 s^{-1}) predicted by the ML models at unit activity of each component in Matrimid polymer (Eq. (12)). The lowest, average, and highest value were from the uncertainty of the ML predictions. The Flory-Huggins interaction parameters of single component at unit activity were calculated by Eq. (6) and Eq. (7).

Component	Lowest sorption (mmol g^{-1})	Sorption in average (mmol g^{-1})	Highest sorption (mmol g^{-1})	Lowest diffusion ($Đ_i^{v,m}$, cm^2 s^{-1})	Diffusion in average ($Đ_i^{v,m}$, cm^2 s^{-1})	Highest diffusion ($Đ_i^{v,m}$, cm^2 s^{-1})
iso-octane	0.510	0.607	0.722	$6.57 \cdot 10^{-9}$	$3.23 \cdot 10^{-8}$	$1.58 \cdot 10^{-7}$
n-octane	1.410	1.540	1.681	$7.02 \cdot 10^{-10}$	$2.22 \cdot 10^{-9}$	$7.07 \cdot 10^{-9}$
methylcyclohexane	1.564	1.677	1.797	$2.91 \cdot 10^{-11}$	$9.84 \cdot 10^{-11}$	$3.32 \cdot 10^{-10}$
toluene	2.703	2.987	3.301	$3.31 \cdot 10^{-8}$	$1.24 \cdot 10^{-7}$	$4.67 \cdot 10^{-7}$
decalin	0.539	0.626	0.727	$1.07 \cdot 10^{-13}$	$2.22 \cdot 10^{-12}$	$4.60 \cdot 10^{-11}$
tert-butylbenzene	1.294	1.508	1.756	$2.67 \cdot 10^{-9}$	$1.37 \cdot 10^{-8}$	$7.11 \cdot 10^{-8}$
iso-cetane	0.182	0.243	0.325	$4.20 \cdot 10^{-13}$	$8.37 \cdot 10^{-12}$	$1.67 \cdot 10^{-10}$
1,3,5-triisopropylbenzene	0.603	0.801	1.062	$5.50 \cdot 10^{-11}$	$2.27 \cdot 10^{-10}$	$9.36 \cdot 10^{-10}$
1-methylnaphthalene	3.192	3.871	4.696	$2.47 \cdot 10^{-12}$	$3.55 \cdot 10^{-11}$	$5.11 \cdot 10^{-10}$

Supplementary Table 5.

Transport parameters of 9 components in the tested mixture in DUCKY-9 polymer predicted by the machine learning (ML) models: sorption uptakes at unit activity (mmol g^{-1}) and Maxwell-Stefan diffusion coefficients ($Đ_i^{v,m}$, cm^2 s^{-1}) by thermodynamically correcting Fickian diffusion coefficients ($D_i^{v,m}$, cm^2 s^{-1}) predicted by the ML models at unit activity of each component in DUCKY-9 polymer (Eq. (12)). The lowest, average, and highest value were from the uncertainty of the ML predictions. The Flory-Huggins interaction parameters of single component at unit activity were calculated by Eq. (6) and Eq. (7).

Component	Lowest sorption (mmol g^{-1})	Sorption in average (mmol g^{-1})	Highest sorption (mmol g^{-1})	Lowest diffusion ($Đ_i^{v,m}$, cm^2 s^{-1})	Diffusion in average ($Đ_i^{v,m}$, cm^2 s^{-1})	Highest diffusion ($Đ_i^{v,m}$, cm^2 s^{-1})
iso-octane	0.063	0.072	0.083	$2.97 \cdot 10^{-8}$	$4.50 \cdot 10^{-8}$	$6.83 \cdot 10^{-8}$
n-octane	0.412	0.475	0.547	$4.11 \cdot 10^{-8}$	$7.26 \cdot 10^{-8}$	$1.28 \cdot 10^{-7}$
methylcyclohexane	0.388	0.437	0.493	$1.68 \cdot 10^{-8}$	$4.49 \cdot 10^{-8}$	$1.19 \cdot 10^{-7}$
toluene	1.848	2.258	2.757	$9.56 \cdot 10^{-8}$	$1.72 \cdot 10^{-7}$	$3.10 \cdot 10^{-7}$
decalin	0.089	0.100	0.112	$4.13 \cdot 10^{-11}$	$7.57 \cdot 10^{-910}$	$1.38 \cdot 10^{-9}$
tert-butylbenzene	0.254	0.302	0.358	$1.35 \cdot 10^{-8}$	$2.16 \cdot 10^{-8}$	$3.47 \cdot 10^{-9}$
iso-cetane	0.028	0.035	0.044	$9.33 \cdot 10^{-10}$	$1.91 \cdot 10^{-9}$	$3.92 \cdot 10^{-9}$
1,3,5-triisopropylbenzene	0.084	0.100	0.120	$1.34 \cdot 10^{-10}$	$3.33 \cdot 10^{-10}$	$8.31 \cdot 10^{-10}$
1-methylnaphthalene	2.579	3.505	4.765	$8.7 \cdot 10^{-10}$	$2.66 \cdot 10^{-9}$	$8.15 \cdot 10^{-9}$

Supplementary Table 6.

Transport parameters of 9 components in the tested mixture in DUCKY-10 polymer predicted by the machine learning (ML) models: sorption uptakes at unit activity (mmol g^{-1}) and Maxwell-Stefan diffusion coefficients ($Đ_i^{v,m}$, cm^2 s^{-1}) by thermodynamically correcting Fickian diffusion coefficients ($D_i^{v,m}$, cm^2 s^{-1}) predicted by the ML models at unit activity of each component in DUCKY-10 polymer (Eq. (12)). The lowest, average, and highest value were from the uncertainty of the ML predictions. The Flory-Huggins interaction parameters of single component at unit activity were calculated by Eq. (6) and Eq. (7).

Component	Lowest sorption (mmol g^{-1})	Sorption in average (mmol g^{-1})	Highest sorption (mmol g^{-1})	Lowest diffusion ($Đ_i^{v,m}$, cm^2 s^{-1})	Diffusion in average ($Đ_i^{v,m}$, cm^2 s^{-1})	Highest diffusion ($Đ_i^{v,m}$, cm^2 s^{-1})
iso-octane	0.079	0.092	0.108	$3.08 \cdot 10^{-8}$	$4.66 \cdot 10^{-8}$	$7.05 \cdot 10^{-8}$
n-octane	0.517	0.597	0.691	$5.53 \cdot 10^{-8}$	$9.55 \cdot 10^{-8}$	$1.64 \cdot 10^{-7}$
methylcyclohexane	0.530	0.602	0.677	$2.33 \cdot 10^{-8}$	$6.06 \cdot 10^{-8}$	$1.57 \cdot 10^{-7}$
toluene	2.510	3.051	3.709	$1.41 \cdot 10^{-7}$	$2.49 \cdot 10^{-7}$	$4.42 \cdot 10^{-7}$
decalin	0.112	0.128	0.146	$7.25 \cdot 10^{-11}$	$1.15 \cdot 10^{-9}$	$1.84 \cdot 10^{-8}$
tert-butylbenzene	0.401	0.476	0.564	$1.72 \cdot 10^{-8}$	$2.78 \cdot 10^{-8}$	$4.49 \cdot 10^{-8}$
iso-cetane	0.036	0.045	0.058	$1.01 \cdot 10^{-9}$	$2.17 \cdot 10^{-9}$	$4.65 \cdot 10^{-9}$
1,3,5-triisopropylbenzene	0.112	0.137	0.169	$1.50 \cdot 10^{-10}$	$3.72 \cdot 10^{-10}$	$9.23 \cdot 10^{-10}$
1-methylnaphthalene	3.618	4.982	6.862	$1.96 \cdot 10^{-9}$	$5.66 \cdot 10^{-9}$	$1.62 \cdot 10^{-8}$

Supplementary Table 7.

Concentrations (mole fractions) of the 12-component mixture feed and permeates (experimentally measured permeate and predicted permeate) separated by Torlon hollow fiber membrane module. Root mean square percentage error (RMSPE) and averaged order of magnitude error (AOME) were calculated by Eq. (14) and Eq. (15), respectively.

Component	Feed	Measured permeate	Std. of measurement	Predicted permeate	Std. Of prediction	RMSPE (%)	AOME
toluene	0.239	0.276	-	0.247	0.007	7.3	0.09
o-xylene	0.05	0.057	-	0.053	0.002		
propylbenzene	0.22	0.234	-	0.225	0.009		
mesitylene	0.08	0.084	-	0.081	0.003		
n-butylcyclohexane	0.08	0.085	-	0.077	0.005		
Tetralin	0.15	0.095	-	0.146	0.033		
1-methylnaphthalene	0.04	0.040	-	0.039	0.014		
biphenyl	0.04	0.034	-	0.037	$7.37\cdot10^{-4}$		
1,3,5-triisopropylbenzene	0.08	0.084	-	0.075	0.004		
dodecylbenzene	0.01	0.007	-	0.009	$7.32\cdot10^{-4}$		
1,3,5-triphenylbenzene	0.01	0.004	-	0.006	$6.37\cdot10^{-4}$		
1,3,5-Tris[(3-methylphenyl)phenylamino]benzene	0.001	$8.44\cdot10^{-5}$	-	$1.72\cdot10^{-4}$	$7.53\cdot10^{-5}$		

*Std. indicates standard deviation.

Supplementary Table 8.

Concentrations (mole fractions) of the 9-component mixture feed and permeates (experimentally measured permeate and predicted permeate) separated by Matrimid TFC membrane. Ideal solution for the unity activity coefficient for all components was assumed (top), and the actual activity coefficients were estimated using PC-SAFT to account for the non-ideal behavior of the mixture (bottom). Root mean square percentage error (RMSPE) and averaged order of magnitude error (AOME) were calculated by Eq. (14) and Eq. (15), respectively.

Component	Feed	Measured permeate	Std. of measurement	Predicted permeate	Std. Of prediction	RMSPE (%)	AOME
iso-octane	0.117	0.081	0.005	0.104	0.001	11.0	0.16
n-octane	0.188	0.174	0.003	0.190	0.001		
methylcyclohexane	0.197	0.213	0.004	0.203	0.001		
toluene	0.327	0.376	0.002	0.347	0.001		
decalin	0.089	0.080	0.001	0.081	0.001		
tert-butylbenzene	0.039	0.045	0.002	0.039	$2.97 \cdot 10^{-5}$		
iso-cetane	0.011	0.002	$5.04 \cdot 10^{-4}$	0.006	$4.40 \cdot 10^{-4}$		
1,3,5-triisopropylbenzene	0.014	0.003	$6.52 \cdot 10^{-4}$	0.012	$2.74 \cdot 10^{-4}$		
1-methylnaphthalene	0.016	0.022	0.002	0.017	$5.09 \cdot 10^{-5}$		

	Activity coefficients at feed	Activity coefficient at permeate	Predicted permeate	Std. Of prediction	RMSPE (%)	AOME
iso-octane	1.17	1.18	0.105	0.001	10.4	0.15
n-octane	0.99	1.00	0.184	$7.34 \cdot 10^{-4}$		
methylcyclohexane	1.23	1.25	0.202	$5.73 \cdot 10^{-4}$		
toluene	1.27	1.25	0.354	$9.79 \cdot 10^{-4}$		
decalin	0.99	0.98	0.080	$5.71 \cdot 10^{-4}$		
tert-butylbenzene	1.00	0.98	0.039	$2.97 \cdot 10^{-5}$		
iso-cetane	0.83	0.83	0.006	$4.49 \cdot 10^{-4}$		
1,3,5-triisopropylbenzene	0.87	0.86	0.011	$2.86 \cdot 10^{-4}$		
1-methylnaphthalene	1.80	1.74	0.019	$8.16 \cdot 10^{-5}$		

*Std. indicates standard deviation.

Supplementary Table 9.

Concentrations (mole fractions) of the 9-component mixture feed and permeates (experimentally measured permeate and predicted permeate) separated by DUCKY-9 TFC membrane. Ideal solution for the unity activity coefficient for all components was assumed (top), and the actual activity coefficients were estimated using PC-SAFT to account for the non-ideal behavior of the mixture (bottom). Root mean square percentage error (RMSPE) and averaged order of magnitude error (AOME) were calculated by Eq. (14) and Eq. (15), respectively.

Component	Feed	Measured permeate	Std. of measurement	Predicted permeate	Std. Of prediction	RMSPE (%)	AOME
iso-octane	0.113	0.097	$1.70 \cdot 10^{-4}$	0.077	$5.65 \cdot 10^{-4}$	4.8	0.05
n-octane	0.170	0.170	$4.02 \cdot 10^{-5}$	0.182	$4.02 \cdot 10^{-4}$		
methylcyclohexane	0.202	0.204	$4.84 \cdot 10^{-4}$	0.214	$5.77 \cdot 10^{-4}$		
toluene	0.325	0.368	$2.09 \cdot 10^{-4}$	0.367	$4.12 \cdot 10^{-4}$		
decalin	0.098	0.085	$3.24 \cdot 10^{-4}$	0.077	$5.60 \cdot 10^{-5}$		
tert-butylbenzene	0.041	0.041	$1.79 \cdot 10^{-4}$	0.041	$1.40 \cdot 10^{-4}$		
iso-cetane	0.014	0.006	$3.98 \cdot 10^{-4}$	0.005	$3.73 \cdot 10^{-4}$		
1,3,5-triisopropylbenzene	0.018	0.009	$3.16 \cdot 10^{-4}$	0.013	$2.92 \cdot 10^{-4}$		
1-methylnaphthalene	0.018	0.02	$6.65 \cdot 10^{-5}$	0.021	$3.37 \cdot 10^{-5}$		

	Activity coefficients at feed	Activity coefficient at permeate	Predicted permeate	Std. Of prediction	RMSPE (%)	AOME
iso-octane	1.17	1.19	0.081	$6.94 \cdot 10^{-4}$	5.5	0.05
n-octane	0.99	1.01	0.194	$4.89 \cdot 10^{-4}$		
methylcyclohexane	1.23	1.28	0.203	$4.49 \cdot 10^{-4}$		
toluene	1.27	1.23	0.381	$4.89 \cdot 10^{-4}$		
decalin	0.99	0.98	0.068	$1.63 \cdot 10^{-4}$		
tert-butylbenzene	1.00	0.87	0.039	$1.24 \cdot 10^{-5}$		
iso-cetane	0.83	0.84	0.004	$2.86 \cdot 10^{-4}$		
1,3,5-triisopropylbenzene	0.87	0.85	0.009	$2.44 \cdot 10^{-4}$		
1-methylnaphthalene	1.80	1.67	0.021	$4.71 \cdot 10^{-5}$		

*Std. indicates standard deviation.

Supplementary Table 10.

Concentrations (mole fractions) of the 9-component mixture feed and permeates (experimentally measured permeate and predicted permeate) separated by DUCKY-10 TFC membrane. Ideal solution for the unity activity coefficient for all components was assumed (top), and the actual activity coefficients were estimated using PC-SAFT to account for the non-ideal behavior of the mixture (bottom). Root mean square percentage error (RMSPE) and averaged order of magnitude error (AOME) were calculated by Eq. (14) and Eq. (15), respectively.

Component	Feed	Measured permeate	Std. of measurement	Predicted permeate	Std. Of prediction	RMSPE (%)	AOME
iso-octane	0.117	0.107	0.003	0.080	0.001	5.9	0.06
n-octane	0.187	0.185	$5.35 \cdot 10^{-4}$	0.197	$5.33 \cdot 10^{-4}$		
methylcyclohexane	0.194	0.196	$2.27 \cdot 10^{-5}$	0.205	$6.99 \cdot 10^{-4}$		
toluene	0.336	0.359	0.007	0.377	$3.89 \cdot 10^{-4}$		
decalin	0.087	0.082	0.002	0.068	$2.68 \cdot 10^{-4}$		
tert-butylbenzene	0.037	0.038	$2.40 \cdot 10^{-4}$	0.039	$4.12 \cdot 10^{-5}$		
iso-cetane	0.011	0.006	$6.94 \cdot 10^{-4}$	0.004	$3.32 \cdot 10^{-4}$		
1,3,5-triisopropylbenzene	0.014	0.009	$8.52 \cdot 10^{-4}$	0.010	$2.57 \cdot 10^{-4}$		
1-methylnaphthalene	0.016	0.017	$1.73 \cdot 10^{-4}$	0.018	$5.27 \cdot 10^{-5}$		

Component	Activity coefficients at feed	Activity coefficient at permeate	Predicted permeate	Std. Of prediction	RMSPE (%)	AOME
iso-octane	1.17	1.19	0.084	0.001	5.7	0.06
n-octane	0.99	1.01	0.192	$6.12 \cdot 10^{-4}$		
methylcyclohexane	1.23	1.28	0.203	$5.71 \cdot 10^{-4}$		
toluene	1.27	1.24	0.378	$8.17 \cdot 10^{-4}$		
decalin	0.99	0.98	0.069	$3.68 \cdot 10^{-4}$		
tert-butylbenzene	1.00	0.97	0.040	$4.71 \cdot 10^{-5}$		
iso-cetane	0.83	0.84	0.004	$3.26 \cdot 10^{-4}$		
1,3,5-triisopropylbenzene	0.87	0.85	0.009	$2.86 \cdot 10^{-4}$		
1-methylnaphthalene	1.80	1.67	0.021	$9.43 \cdot 10^{-5}$		

*Std. indicates standard deviation.

Supplementary Table 11.

Feed concentrations and solvent properties of methanol and guaiacol. δ_D, δ_P, and δ_H are Hansen solubility parameters for dispersion, polarity, and hydrogen-bonding each.

Component	Hansen solubility parameter ($MPa^{0.5}$)			Vapor pressure (torr)	Molar volume (cm^3 mol^{-1})
	δ_D	δ_P	δ_H		
methanol	14.7	12.3	22.3	94	40.46
guaiacol	18	7	12	0.103	111.84

Supplementary Table 12.

Transport parameters of methanol and guaiacol in DUCKY-9 polymer predicted by the machine learning (ML) models: sorption uptakes at unit activity (mmol g^{-1}) and Maxwell-Stefan diffusion coefficients ($Đ_i^{v,m}$, cm^2 s^{-1}) by thermodynamically correcting Fickian diffusion coefficients ($D_i^{v,m}$, cm^2 s^{-1}) predicted by the ML models at unit activity of each component in the DUCKY-9 polymer (Eq. (12)). The lowest, average, and highest value were from the uncertainty of the ML predictions. The Flory-Huggins interaction parameters of single component at unit activity were calculated by Eq. (6) and Eq. (7).

Component	Lowest sorption (mmol g^{-1})	Sorption in average (mmol g^{-1})	Highest sorption (mmol g^{-1})	Lowest diffusion ($Đ_i^{v,m}$, cm^2 s^{-1})	Diffusion in average ($Đ_i^{v,m}$, cm^2 s^{-1})	Highest diffusion ($Đ_i^{v,m}$, cm^2 s^{-1})
methanol	7.41	9.26	11.75	$1.02 \cdot 10^{-7}$	$2.39 \cdot 10^{-7}$	$5.61 \cdot 10^{-7}$
guaiacol	1.91	2.36	2.88	$4.58 \cdot 10^{-9}$	$1.32 \cdot 10^{-8}$	$3.81 \cdot 10^{-8}$

Supplementary Table 13.

Concentrations (mole fractions) of the binary biofuel mixture feed and permeates (experimentally measured permeate and predicted permeate) separated by DUCKY-9 TFC membrane. Ideal solution for the unity activity coefficient for all components was assumed (top), and the actual activity coefficients were estimated using SR-POLAR model to account for the non-ideal behavior of the mixture (bottom).

Component	Feed	Measured permeate	Std. of measurement	Measured total flux (L m^{-2} hr^{-1})	Predicted permeate	Std. Of prediction	Measured total flux (L m^{-2} hr^{-1})
methanol	0.8	0.822	0.002	4.88 ± 0.4	0.821	$8.40 \cdot 10^{-5}$	3.69 ± 2.4
guaiacol	0.2	0.178	0.002		0.179	$8.40 \cdot 10^{-5}$	

Component	Activity coefficients at feed	Activity coefficient at permeate	Predicted permeate	Std. Of prediction	Measured total flux (L m^{-2} hr^{-1})
methanol	0.97	0.98	0.823	$2.86 \cdot 10^{-4}$	2.81 ± 1.85
guaiacol	0.78	0.75	0.177	$2.86 \cdot 10^{-4}$	

*Std. indicates standard deviation.

Supplementary Table 14.

Root mean square percentage error (RMSPE) and averaged order of magnitude error (AOME) analysis on the concentration predictions of Permian crude oil fractionation by SBAD-1 membrane.

	RMSPE (%)	AOME
Overall	6.8	0.20
Under 100 °C	6.2	0.12
100 °C – 200 °C	4.0	0.06
200 °C – 300 °C	5.3	0.11
300 °C – 400 °C	6.7	0.19
400 °C – 500 °C	7.4	0.25
500 °C – 600 °C	8.0	0.31
Above 600 °C	3.9	0.15

Supplementary Table 15.

Root mean square percentage error (RMSPE) and averaged order of magnitude error (AOME) analysis on the concentration predictions of Arabian Light crude oil fractionation by DUCKY-9 membrane.

	RMSPE (%)	AOME
Overall	5.9	0.12
Under 100 °C	4.1	0.05
100 °C – 200 °C	1.3	0.02
200 °C – 300 °C	3.2	0.06
300 °C – 400 °C	5.6	0.12
400 °C – 500 °C	6.5	0.15
500 °C – 600 °C	7.8	0.18
Above 600 °C	11.7	0.36
